# Improving treatment adherence for blood pressure lowering via mobile phone SMS-messages in South Africa: a qualitative evaluation of the SMS-text Adherence SuppoRt (StAR) trial

**DOI:** 10.1186/s12875-015-0289-7

**Published:** 2015-07-03

**Authors:** Natalie Leon, Rebecca Surender, Kirsty Bobrow, Jocelyn Muller, Andrew Farmer

**Affiliations:** Health Systems Research Unit, South African Medical Research Council, P.O. Box 19070, Fransie Van Zyl Drive, Tygerberg, Cape Town, 7505 South Africa; Department of Social Policy and Intervention, University of Oxford, 32 Wellington Square, Oxford, OX1 2ER UK; Chronic Disease Initiative for Africa, Division of Diabetes and Endocrinology, Department of Medicine, University of Cape Town and Groote Schuur Hospital, Main Road, Observatory, Cape Town, 7925 South Africa; Nuffield Department of Primary Care Health Sciences, University of Oxford, Gibson Building, OX2 6GG Oxford, UK

**Keywords:** Mobile phone-based SMS-messages, mHealth, Blood pressure treatment, Hypertension, Adherence, South Africa, low-and middle-income countries, primary-care, qualitative methodology, patient perspective

## Abstract

**Background:**

Effective use of proven treatments for high blood pressure, a preventable health risk, is challenging for many patients. Prompts via mobile phone SMS-text messaging may improve adherence to clinic visits and treatment, though more research is needed on impact and patient perceptions of such support interventions, especially in low-resource settings.

**Method:**

An individually-randomised controlled trial in a primary care clinic in Cape Town (2012–14), tested the effect of an adherence support intervention delivered via SMS-texts, on blood pressure control and adherence to medication, for hypertensive patients. (Trial registration: ClinicalTrials.gov NCT02019823). We report on a qualitative evaluation that explored the trial participants’ experiences and responses to the SMS-text messages, and identified barriers and facilitators to delivering adherence support via patients’ own mobile phones. Two focus groups and fifteen individual interviews were conducted. We used comparative and thematic analysis approaches to identify themes and triangulated our analysis amongst three researchers.

**Results:**

Most participants were comfortable with the technology of using SMS-text messages. Messages were experienced as acceptable, relevant and useful to a broad range of participants. The SMS-content, the respectful tone and the delivery (timing of reminders and frequency) and the relational aspect of trial participation (feeling cared for) were all highly valued. A subgroup who benefitted the most, were those who had been struggling with adherence due to high levels of personal stress. The intervention appeared to coincide with their readiness for change, and provided practical and emotional support for improving adherence behaviour. Change may have been facilitated through increased acknowledgement of their health status and attitudinal change towards greater self-responsibility. Complex interaction of psycho-social stressors and health service problems were reported as broader challenges to adherence behaviours.

**Conclusion:**

Adherence support for treatment of raised blood pressure, delivered via SMS-text message on the patient’s own phone, was found to be acceptable, relevant and helpful, even for those who already had their own reminder systems in place. Our findings begin to identify for whom and what core elements of the SMS-text message intervention appear to work best in a low-resource operational setting, issues that future research should explore in greater depth.

**Electronic supplementary material:**

The online version of this article (doi:10.1186/s12875-015-0289-7) contains supplementary material, which is available to authorized users.

## Background

High blood pressure is a leading modifiable risk factor for cardiovascular and related diseases and is associated with more than 9 million deaths a year worldwide [1], with eighty per cent of the global burden of disease being in low and middle-income countries [1, 2]. Sub-optimal adherence to prescribed treatments is associated with an increased risk of blood-pressure-related complications and mortality [3]. Missed appointments for collection of medicine and doctor’s appointments and challenges with taking lifelong treatment are amongst the major reasons for sub-optimal adherence, leading to poor patient outcomes and additional costs on the health services [4]. Interventions delivered via mobile-phone SMS-text messaging have the potential to improve adherence to clinic visits and treatment, though more research is needed on the impact and on patient and provider perceptions of such interventions to further develop and inform policy [5]. Research is also needed on the up-scalability of such interventions within mainstream health services especially in low and middle-income settings [6–8].

We report here on a qualitative evaluation of the patient experiences of a novel adherence support intervention delivered via the patient’s own mobile phone in South Africa. The high prevalence of mobile phone ownership and use in South Africa offers a setting in which it is possible to use them to improve delivery of health-care in a developing country context. Although formally classified as a middle-income country, the historical and widespread poverty and particular patterns of racial and geographical inequality means that human development indices for South Africa as a whole are significantly lower than for other countries with similar income levels, and key morbidity and mortality health indicators are more typical of a low income country [9].

A large three-armed randomized controlled trial, (the SMS-text message for adherence support or StAR trial), tested the effectiveness of an adherence support intervention delivered via SMS-text message on blood pressure and treatment adherence, compared to usual care in the general outpatient population of adults with high blood pressure attending a single, large, public sector primary health care clinic in Cape Town, South Africa.

The qualitative evaluation investigated the wider potential for health interventions delivered via mobile phone by exploring patients’ experience of the trial, including use, interpretation and response to SMS-text messages, as well as barriers and facilitators to delivering treatment support via SMS-text message.

## Method

### Setting and population

The trial took place among the general adult population attending the outpatient chronic disease services in a single large public sector clinic in Cape Town, South Africa. This facility provides comprehensive primary health care services to an ethnically diverse, socio-economically deprived population of about 100 000 people from two communities: based on 2011 census data population ethnicity is mostly black African (51 %) or mixed ancestry (48 %), about one third of households live in informal dwellings or shacks, and 64 % of households have a monthly income of less than $270.00 [10]. The clinic is roughly within walking distance of both the communities. As for all primary care facilities in South Africa, all health care services and medicines are provided free of charge. In the chronic disease services, stable patients on treatment are reviewed by a clinician (doctor or prescribing nurse) every three- to six-months although patients initiating treatment for the first time or those considered poorly controlled are reviewed more frequently. At clinical review patients are prescribed medication on repeat until their next scheduled review. Medicines are dispensed in 28 day increments, which patients collect from the on-site pharmacy. As part of a strategy to reduce clinic waiting times the local department of health has introduced a service whereby patients on a stable regimen with a valid prescription on repeat (for up to six-months) receive their monthly supply of medicines pre-packaged from the “chronic dispensing unit” (CDU), a centralised service, which are supplied to the clinic pharmacy 48 hours before a patients’ scheduled collection date. Medicines not collected are returned to the CDU 72 hours after the scheduled appointment.

The qualitative evaluation study of patient experiences was carried out as the trial was being drawn to a close with most participants having already concluded their participation in the trial.

The trial is registered at clinicaltrials.gov (NCT02019823). The trial is also registered with the South African National Clinical Trials Register (SANCTR DOH-27-1212-386) and the Pan Africa Trial Register (PACTR201411000724141). In Box 1, we provide details of the trial intervention and design. The trial protocol is reported in a separate paper [11] and the trial intervention development and trial results will be reported in future publications.

### Study design

We employed a qualitative design using focus groups and in-depth interviews. There is growing awareness that in-depth qualitative research for understanding the patient experience is valuable in redressing some of the methodological limitations of randomised controlled trials [12]. The design benefitted from combining the high-face validity produced from the broad discussion and insights resulting from the focus group interaction, with the opportunity for more in-depth targeted discussion in follow up individual interviews [13]. We developed a research framework to explicitly theorise connection between the research objectives of the quantitative trial and the objectives of qualitative patient study and this formed the conceptual backdrop that guided the analysis of the results. (See Additional File 1: Figure S1. Research framework for StAR trial qualitative study).

### Sampling

We used a combination of convenience and purposive sampling to recruit participants from the trial during the trial follow-up period. We hosted two focus groups of trial participants who met our general criteria for diversity of age, language and ethnic background. This was  in order to explore general aspects of the intervention and to test our sampling strategy. In order to explore in detail the variety of perceptions and experiences expressed in the focus groups we conducted additional in-depth interviews. We explored the views of twenty-two trial participants in two focus groups (of 11 participants each) and carried out fifteen individual in-depth interviews, with eleven of the interviewees being drawn from the focus groups.

### Data collection

An independent research team comprised of researchers from the South African Medical Research Council (NL and JM) and Department of Social Policy and Intervention, Oxford University (RS), led the qualitative study. The qualitative research team collaborated closely with the trialists in the research design stage and interpretation of findings, but was solely responsible for data collection, final data analysis and interpretation of qualitative data.

Data were collected between March and May 2014, using a single semi-structured questionnaire, which was adapted during the data collection process. Questions in the group and individual interviews focussed on understanding the patient’s experience of chronic illness, their adherence behaviour in general, their experience and perception of the intervention, including their comfort with SMS-text message delivery technology and the effect if any, of the intervention on their adherence behaviour, health and wellbeing. Focus groups and interviews were conducted in English in private spaces in the clinic setting, digitally recorded and transcribed. Data collection stopped when data saturation was reached, that is, when it was judged that no significant new data would emerge from further focus groups and interviews.

### Data analysis and ethics

Data analysis was carried out iteratively, with concurrent data collection and analysis. This allowed for shifts in further data collection from focussing on the responses to specific aspects of the intervention (such as the types of individual SMS text-message), to effects and impact and later, exploring more distal effects, such as patient wellbeing. We used comparative analysis and thematic analysis techniques. The main tools were three sets of independently coded summaries; the initial post-interview summaries of the interviewer (JM) and the coded summaries of full transcripts (NL, RS). Themes were developed in relation to the key research questions about patient experience and effect of the SMS-text messages and in reference to the research framework we designed (see Additional file 1: Figure S1). Data analysis considered themes that both ran across different individual interviews and themes within individual interviews, the latter allowing the tracking of reported change in behaviour for individual patients. Uncommon or oppositional responses were discussed and a potential explanation for the findings sought in the data.

Credibility of the findings was enhanced through iterative data collection, the use of multi-method design (focus groups and in-depth interviews) and the triangulation of the analysis by three qualitative researchers [14]. The collaborative sharing of interim findings between the quantitative and qualitative teams provided opportunity for feedback and scrutiny and to generate and deepen insights that informed the interpretation of the findings [15, 16].

This study was approved by the Human Research Ethics Committee of the University of Cape Town (HREC UCT 017/2014), the Oxford Tropical Research Ethics Committee (OXTRE 13–14), and received governance approval from the Cape Town Metro District Health Services, Western Cape Province. All the requirements of the Helsinki Declaration of 2008 were fulfilled. Informed consent was obtained in writing from all participants and any information that might allow individuals to be identified has been deleted to ensure their anonymity. In lieu of a meal at the interview, participants were provided with a food voucher and transport costs.

## Results

### Demographics

Of the twenty-two participants in the two focus groups, 16 were females and 6 males, with an age range of 36 to 78 years old. Of the 15 participants interviewed (eleven of whom were drawn from the focus groups), 8 were female and 7 male, with an age range of 45 to 78 years. Participants had a similar age range and socio-economic characteristics to the general population of trial participants (poor socio-economic circumstances and high levels of unemployment).

### General experience of StAR trial

The overall responses to the SMS-text message-based intervention and participation in the trial were positive. A few reported initial ambivalence about participating in the trial (fearing it would increase already lengthy waiting time in the clinic), and others felt they already had reminder systems in place, but they nevertheless praised and appreciated the usefulness of the text messages.

The majority of participants were comfortable with the technology of accessing and reading their SMS-text messages and reported no technical difficulties with receiving messages. A few participants reported difficulties or unease with the technology; two said they were not active or confident users of their phones, one complained of interrupted mobile phone reception, one found it inconvenient to receive SMS-messages during work time and another reported a problem with the alignment of reminder dates which was later corrected. One participant suggested that voice-mail should be considered for those who cannot read. There was an appreciation for having a choice of languages for the SMS content.

In response to questions about their experience of the SMS-text message intervention, participants typically differentiated two main components of the research process; the SMS-text messages received on their personal phones and the trial-specific follow-up visits to measure blood pressure. The trial clinic site (for administration and follow-up visits) was situated in a temporary structure that was erected in the clinic courtyard, and referred to by participants as the ‘StAR Tent' or simply, the ‘Tent’. Positive experience of the SMS-texts and positive interactions with trial staff in the StAR Tent were often intertwined, making it difficult at times to separate out participant responses to the SMS-text messages alone. Nevertheless when asked how they experienced the SMS-text messages, what they remembered of the content, and what if any, they found most valuable, participants identified a range of benefits associated with the SMS-text message intervention.

### Effects of the SMS-text intervention on attitudinal and behaviour change

Whilst most patients had a positive experience of the SMS-text intervention, not all reported that it helped improve their adherence behaviour. They reported that their reminder systems had been working well for the most part, with only occassional forgetting to collect their medicine. A smaller group (about one third), found the intervention highly beneficial. This sub-group reported that it resulted in improved attitude to their health condition, and improved adherence behaviour and health.

#### Changes in acknowledgement of disease status and attitude

In general (not only in the subgroup with reported behaviour change), participants reported an increase in understanding the seriousness and life-long nature of their disease and the need to make improvements to their health practices. Those who reported benefitting the most, found the SMS- text messages with tips for healthy living particularly useful. They noted that the SMS-text messages increased their awareness about the need to take more responsibility for managing their own health. For instance, Zola, a musician, described how he had been struggling to be “disciplined” about his healthcare and how the text-messages made him rethink his attitude to adherence.

“Before the SMS I was an outlaw…I used to take it [medication] any time…The doctors tell me, but I didn’t care what happens… I didn’t have that discipline, but the SMS gave me discipline you know. Yeah it takes you out of that ‘outlaw’ thing you know, it disciplines you… to bring you back and say, ‘Aye, be careful’…” (Zola, 67)

Several participants described how they became more resolved about living a healthier lifestyle and taking small steps towards better health practices.

“I’m not eating too much salt as the StAR told me, and the sugar. Also not coffee, and Coke [Coca Cola] I reduced myself, but I like Coke. But I try not to take it too much…And they give advice that you must have exercise in your body. Yeah, so that is why we make exercise in the morning with my little ones.” (Nomsa, 45)

#### Developing more robust reminder systems

As mentioned earlier, several  participants reported that they considered themselves to be adherent to collecting the medication and to using it correctly, and therefore did not feel a strong need for additional reminder systems. Several of these participants nevertheless acknowledged having occasional lapses in adherence and that they appreciated the extra reminder support provided by the SMS-text messages. Those who acknowledged having weak or no reminder systems reported that the SMS- text message reminders were key to them improving their adherence. The messages helped them become more organized and to developed more robust reminder systems.

“… I was so pleased because sometimes really, then I do forgot to take tablets… then your phone will ring and remind you that ‘Don’t forget to take your tablets’ and ‘You don’t forget to come to the clinic on such a date.’” (Lindiwe, 62)

The timing and format of the reminders seemed particularly useful; especially receiving a reminder two days before (allows one to prepare in advance) and the notification of a missed appointment (encouragement and reminder that the pre-packed medication can still be collected for two days after the missed appointment).

…I like these messages because it tells you in advance so that you prepare yourself, that on this date you gonna come get your medicine. It also tells you to take care of your health… to look after your health. But before, nobody cared what I did or what I don’t do.” (Melanie, 45)

Participants also reported putting in place practical measures to improve their adherence by following advice they received via the SMS-text messages, such as for example, getting family to help with reminders and using a pill-box to help with regular taking of medicines.

“You even plan your medicine beforehand because that’s what they teach us. To have a [pill] box daily, like today, tonight, today, tonight…Sometimes you forget, okay, … ‘Did I take my medicine or didn’t I?’ …and then you go to the box and you find out ‘No, that section has still got that medicine’. Then you know ‘Oh I didn’t’, then I just take it. You can’t overdose yourself and you can’t sort of forget about it.” (Zola. 67)

Two participants reported using the function that allowed one to reschedule appointments electronically, via a free return SMS (interactive intervention arm only). Unexpected family illness in the one case and an unpredictable work schedule in another, led to participants changing appointment dates.

“I’m a musician so there are times that I have to clash with my dates… then I just sort of phone them [changing via SMS-text] and they used to organise my medicine and then I come the following day and then just pick up the medicine without going through the procedure of skipping a day [missing an appointment].” (Zola, 67)

These patients reported finding it very difficult to change appointments within the normal clinic system, i.e. outside their trial participation, (unable to reach the clinic telephonically or staff refusing requests) and they welcomed this ability for more control and flexibility.

#### Reinforcement of positive change

There was a perceived long-term impact for several participants who reported benefitting more from the intervention; they described their methods for routinising and sustaining their new, improved  adherence behaviours. For instance, Zola (the musician quoted earlier), noted that the SMS- text messages became “embedded in me” and like Ferdi (quoted below), he continued to be more organized about his health after the trial ended. Ferdi developed his own time-table and continued with using a pillbox and weighing himself after the trial ended.

“Yes I’m very much aware and take responsibility of myself because at first I didn’t. I wasn’t aware, but they taught me how to be aware, how to take my medication and so, when that sinked into me and when I practiced it every day, it became a part of me. And I don’t think it’s gonna go out of me because it’s now part of me… I take my tablets, I weigh myself in our bathroom, I look at my weight… and everything.” (Ferdi, 49).

Others also reported notable improvements in health linked to adherence behaviour changes. For instance, Nomsa, reported feeling healthier (less fatigue and fewer headaches) since taking her medication regularly, implementing dietary changes and being more active (doing housework and playing with her daughter). She called her trial experience “life-saving” and hinted at sustained positive changes in adherence.

“My experience with the StAR…changed my life …Because sometimes I forgot to take my tablets, but because of their messages and reminding me all the time, I didn’t forgot now at all… I know that even now when I’m not getting the SMS-ses, I’m fine because as from that time, everything is working right in my mind.” (Nomsa, 45)

This sentiment was echoed by others; another participant indicated that her new reminder system became part of a lasting shift in attitude or “mentality”.

“StAR did try to build that mentality of taking my medication as prescribed and every day, and not to forget to eat healthy. You know it’s always there and it started to be in my ears always.” (Dina, 56)

There was no indication of ‘message-fatigue’ or complacency (i.e. that people got bored, irritated or indifferent) about receiving text-messages on an ongoing bases (about one message a week for 12 months including monthly reminders to collect medication). In fact the opposite appeared to be the case, with many saving and re-reading the messages and wanting to share their messages, and advise others.

#### Social connectedness and motivation

Participants valued the supportive content and polite tone of the text-messages, together with the supportive demeanour of the trial staff. This appeared to have generated a sense of being recognised, respected, valued and cared for. Participants likened their trial experience to a partnership and valued “being listened to” and being treated “as equals”. Small gestures during visits to the StAR Tent for example, trial staff  asking after a patient’s health and wellbeing, addressing them by name and praising them for making efforts to improve their health, were all appreciated by participants. Text messages signed by a named provider (for example, the lead doctor or pharmacist), and receiving a personalised ‘Happy birthday’ SMS-text message touched and delighted participants. It reportedly made them feel “special” and “cared for” and appeared to reinforce an impression of personalised care. This relational aspect of the trial experience seemed to make it easier for participants to listen and respond positively to health advice dispensed by the SMS-text messages.

“…I just think, yoh!, I’m so special to them because they give care that I did not get before. You feel free in your heart once you see someone is taking care of you.” (Nomsa, 45)

And

“…the special message on my birthday that morning… it was very impressive. I felt so good and no presents could make my day because the message made my day.” (Delia, 64)

Participants often contrasted their positive interactions with trial staff with their negative perceptions of standard clinic care. Nomsa, like many others, contrasted ‘feeling cared for’ as she experienced in the trial, with a her sense of a lack of care in her normal clinic experience. She hinted at the importance of feeling supported, for promoting adherent behaviour.

“When I joined StAR…they give me advice. ‘[It’s] important to take your tablets because if you don’t take your tablets you will get a stroke…’ But also the [clinic] nurses told me about that…I don’t know why I don’t listen to them… But from the StAR, I get someone to stand next to me and show me the dangers I put myself in”. (Nomsa, 45)

#### Readiness for change

Participants who appeared to benefit the most, described how major life stressors became the backdrop for readiness to change adherence behaviour. They indicated that the timing of the trial intervention provided a ready-made opportunity to change not only their adherence behaviour, but to tackle their other stressors more positively. For example, a participant who had just emerged from an abusive relationship and divorce, described how her depression and self-blame (for the abuse and for acquiring a chronic disease) resulted in her stopping her blood pressure lowering medication, “as a way of punishing myself”. Through her participation in the trial she felt valued by others and this she said, restored her confidence and “nudged” her in the direction of better self-care.

“It gave me the confidence being with StAR, to take an HIV test which I was scared for and lots of other tests. … they kind of nudged me into caring *more* for myself, you know?” (Sara, 55)

This sentiment was shared by several of the participants who benefitted the most: they described a connection between their trial participation and their motivation for positive life changes. Another example illustrating how readiness for change coincided with opportunities for change through trial participation, is provided by a participant who was recovering from recent cancer treatment. He viewed his cancer diagnosis as a “wake-up call” to take better care of his health and ascribed his good recovery (from cancer), to learning through the STAR trial, how to take better care of his health.

“…I usually forgot to take my tablets because there was no reminder…So for me, they gave me a whole different perception of what I had before I met them…. I can salute them because they taught me how to do it, how to look after myself. That’s why I’m so proud… (Ferdi, 49).”

### Factors affecting adherence

In the course of narrating their experience of the trial, patients identified a range of factors that influenced their adherence/non-adherence behaviours. These factors were not directly related to receiving the SMS- text message intervention, but are worth describing here as they illuminate how interaction between psychosocial and health service factors influence sub-optimal adherence. It therefore provides the broader context against which the SMS- text intervention should be viewed and generates insights into other facilitators and barriers to changing adherence behaviour.

#### Psycho-social factors

Participants commonly described how stressors of daily living made it harder to pay attention to their personal health needs and to adhere to treatment. Stressors included poverty and material deprivation, emotional stress due to competing demands of caregiving roles, bereavement, job loss, interpersonal violence, and living in fear, associated with high levels of neighbourhood crime. These stressors, together with discomfort from perceived side effects of medication or personal and community beliefs about alternative medicines, often resulted in use of alternative medicines, using medication irregularly or “taking a break” from their medication to “cleanse” the body.

“I just jump [skip] it [medicine] because I can see this [side effects] is coming from these tablets… As soon as I eat my breakfast I feel sleepy. What is that? You’re sleepy in the morning… ‘No, it is this tablets.’” (Henry, 78)

#### Health service organization factors

Participants commonly described a range of negative experiences with the existing health services and explained how this frequently influenced non-adherent behaviour. Reported problems include long waiting times, frequent and unexplained changes to patient flow systems, unresponsive clinical care, unprofessional health worker practices (being scolded and belittled) and inefficiency (“missing” patient folders”) and medicine stock-outs. Participants felt particularly aggrieved by unfair “labelling” of patients as “non-adherent” especially as this was associated with negative treatment of patients (scoldings, threats to withdraw patient care and “punishment” in the form of deliberately prolonging patient waiting time).

“Even if you explain…sometimes you’re very sick, you’ve got flu, and you can’t come [to one’s appointment]. They don’t want to understand that, they will punish you. [When you do attend again] You will sit here from the morning without being attended to…” (Dina, 56)

Participants reported that in response to these health service problems and to avoid negative treatment by clinic staff, they sometimes attended clinic irregularly, rationed and stock-piled their medication and “lied” about their adherence to medication, all in an effort to avoid negative interactions at the clinic.

## Discussion

There are few examples of large-scale mobile phone-based interventions implemented in mainstream health services and even fewer evaluate patient experiences. This work has been driven by a need to understand the process and wider impact of delivering adherence support via mobile phones in a developing country context, and the need for enhanced methodologies for evaluating such interventions.

Adherence support for treatment of raised blood pressure, delivered via SMS-text message on the patient’s own phone, was found to be acceptable, relevant and helpful to a broad range of participants, even for those who already had their own reminder systems in place. Most participants across a wide age range (36–78 years) were comfortable with the basic technology of voice and SMS-text functions of their mobile phone and accessed their SMS-text messages with ease.

The findings identified a sub-group of patients who appeared to benefit more than others from the SMS-text message intervention. These participants acknowledged being vulnerable due to various life stressors, and recognised their personal struggles with adherence to high blood pressure treatment. Several experienced their participation in the trial as coming at the right time for making positive life changes. It would seem that the SMS-text message intervention provided a ready-made opportunity and mechanism to support a positive change in their adherence behaviour.

For the sub-group of participants who reported adherence behaviour change, there are indications that the intervention may have operated in multiple ways to facilitate change. On a cognitive-behavioural level, the SMS-text messages increased disease awareness, provided health tips and helped to develop and reinforce more robust reminder systems. Through a combination of supportive SMS content and positive interactions with trial staff, the intervention also appeared to provide emotional support (generating a sense of social connectedness and being cared for) that may have motivated increased self-responsibility for change. Similar feelings of social support was engendered by receipt of SMS-text messages and phone calls, in the Kenyan WelTel study [17], which was carried out in another low-resource setting.

Specific elements of the SMS-text messaging relating to content, tone and timing, also contributed to positive appraisal of the intervention. The polite and respectful tone and use of personalised SMS-text messages (e.g. acknowledging birthdays, named health providers) were singled out for generating a sense of being respected and valued. Practical health advice was another valued component, and the interactive function (changing appointments via SMS).

Patients balanced multiple stressors related to their personal, family, socio-economic circumstances and health systems barriers, and reported a range of associated intentional and unintentional non-adherence behaviours that matches those reported in previous studies [18, 19]. Non-adherent behaviours included irregular clinic attendance, ignoring clinical advice, not taking medication as prescribed, self-dosage, intermittent use of medication, treatment breaks and “stock-piling” medication in anticipation of shortages. Studies have documented similar underlying psycho-social and health service reasons to those found in this study [18] many of which may not be easily modifiable via adherence change interventions that focus on information and communication technology only.

There are indications of similarities in patient health beliefs across different cultures and settings [19] which may mean that for SMS-text messages to be helpful, the content may not need to be radically different between settings. However, the success of a fully integrated SMS-text message intervention may invariably be dependent on the overall functioning and responsiveness of the health services, as this emerged as an important influence on adherence. Participants highlighted the importance of feeling cared for and respected as patients and the need for health staff to have tolerance of patients’ struggles to achieve good adherence.

A recent systematic review of trials of mobile phone based interventions has examined the factors that might be associated with their effect [8], and found similarly that acceptability and reliability of the technology were key issues. The review also identified a number of issues as problematic, though these were not evident in our study. Patient ‘fatigue’ with messages did not appear to be a problem. There was also no gender difference as reported elsewhere [20], with men and women both having unrestricted access to mobile phones. Privacy concerns were also not raised, perhaps due to greater levels of individual ownership rather than shared handsets, and the fact that SMS-messages did not contain sensitive personal health information.

A range of social cognition models have been proposed to underpin interventions to improve adherence (including the theory of planned behaviour [21], and cognitive behavioural approaches [22]), but the focus on a single behavioural or cognitive target for adherence interventions (such as volition or motivation), may account for the failure of many adherence interventions to bring about sustained behaviour change [23]. Furthermore, this qualitative analysis sheds light on the fact that sometimes non-adherence is a result of factors very difficult or impossible to control, and that patient self-care can suffer when a person perceives him or herself to be penalized for their suppossed non-adherence. This study therefore highlights the need to understand the impact of SMS-text messages embedded in a larger environment of adherence support that is coupled with understanding of the complex factors influencing non-adherence and where there is understanding of and tolerance for occasional lapses in adherence.

### Strengths and limitations of the study

The analysis was carried out independently of knowledge of the trial results and to which intervention group within the trial patients were allocated, thus allowing for greater level of objectivity. On the other hand, it meant we could not focus in on explaining possible underlying reasons for particular trial outcomes. Interviews were carried out as the trial was drawn to a close, so some may have had difficulty with recalling details of their initial trial experiences over the past 12 months. The overwhelming gratitude for the positive interactions with trial staff which was in strong contrast to patients’ usual care experiences, raises the question of social desirability of responses. However, whilst the majority of participants were positive about receiving SMS-text messages, fewer participants reported positive behaviour change, making it less likely those reports were due to social desirability. In addition, poor responsiveness of public sector health services in South Africa have been well recorded [24, 25]. Whilst the  aspect of feeling cared for, respected and valued played a major role in this study, especially as contrasted to usual care, this response may not be generalizable beyond low resource settings.

A strength of the qualitative evaluation is that the qualitative team was independent from the team conducting the trial, which promoted objectivity. There was collaboration between the qualitative and the quantitative research teams on the interpretation of findings and robust engagement increased meaningfulness and credibility of the findings. Acknowledgement of the different roles and perspective of team members is recommended in process evaluations of trials [16].

## Conclusion

Adherence support for treatment of raised blood pressure, delivered via SMS-text message on the patient’s own phone, was found to be acceptable, relevant and helpful. In addition, our findings begin to identify for whom and what core elements of the SMS-text message intervention appear to work best in an operational setting, issues that future research should explore in greater depth.

This work has a number of broad implications. It highlights the importance of both the practical utility and relational aspects of an SMS-text message adherence intervention in an operational setting, and the potential for it to improve adherence behaviour. It confirms the value of a qualitative evaluation component in a behaviour change trial, for generating insights about patients’ experience and possible causal pathways of behaviour change. There may be further advantages to conducting qualitative evaluations throughout a trial, to examine more closely the facilitators and barriers encountered during trial implementation and tracking patient experience over time. Finally, the study highlighted the need to underpin future mobile phone-based adherence interventions with behaviour change theories that take cognisance of the complex mix of psychosocial and health service influences on adherence behaviours [26].

## Additional file

Additional file 1: Figure S1. Research framework for StAR trial qualitative study.
