# Supplementary figures and images for: Improving treatment adherence for blood pressure lowering via mobile phone SMS-messages in South Africa: a qualitative evaluation of the SMS-text Adherence SuppoRt (StAR) trial
Source: BMC Fam Pract. 2015 Jul 3;16:80. doi: 10.1186/s12875-015-0289-7 (PMC4490665; doi:10.1186/s12875-015-0289-7)

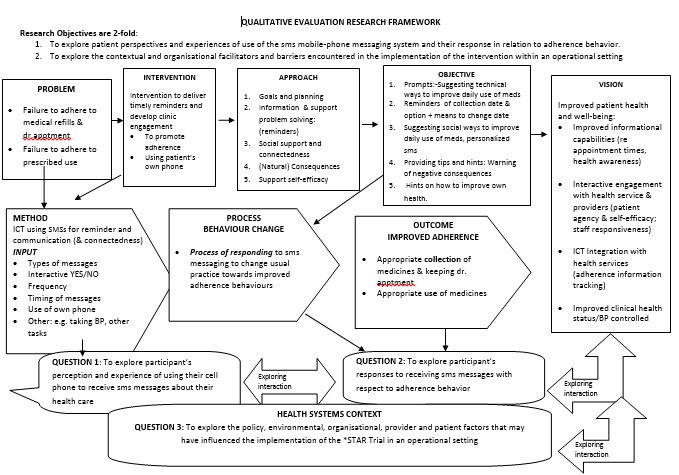

Supplement: Additional file 1: Figure S1. — Research framework for StAR trial qualitative study. [file 12875_2015_289_MOESM1_ESM.png]
